# Editorial: Acute Promyelocytic Leukemia — Towards A Chemotherapy-Free Approach to Cure in All Patients

**DOI:** 10.3389/fonc.2021.831308

**Published:** 2022-01-20

**Authors:** Harinder Gill, Yok-Lam Kwong, Farhad Ravandi

**Affiliations:** ^1^ Department of Medicine, The University of Hong Kong, Hong Kong, Hong Kong SAR, China; ^2^ Department of Leukemia, University of Texas MD Anderson Cancer Center, Houston, TX, United States

**Keywords:** acute promyelocytic leukemia, arsenic trioxide, chemotherapy-free, tetraarsenic tetrasulfide, oral arsenic trioxide

Acute promyelocytic leukemia (APL) is a subtype of acute myeloid leukemia (AML) characterized by t(15;17)(q24;21) and the fusion gene *PML-RARA*. With optimal supportive care and frontline use of all-trans retinoic acid (ATRA) and chemotherapy, first complete remission (CR1) rates of more than 90% and long-term survival of more 80% can be achieved. Regimens that include As_2_O_3_, ATRA and chemotherapy result in a CR rare of 90-100% and OS between 86-97% (1–3). Frontline treatment of APL has evolved rapidly. An emerging theme is the incorporation of As_2_O_3_ early in the treatment algorithm, starting from induction to consolidation.

Various forms of arsenic were used in China for over 5000 years. Arsenic first appeared in Western Medicine in the eighteenth century. In hematology, oral arsenic was first reported in the treatment of chronic myeloid leukemia from the 1860s to 1920s in Germany and Boston City (4). This treatment was phased out following World War II with the development of alkylating chemotherapy and radiotherapy. Intravenous pure As_2_O_3_ solution was first used in Harbin, China in 1973. Its mechanism of action, pharmacokinetics and clinical efficacy was extensively published in 1996. In this Research Topic collection, Kumana et al. gave a historical account on the development of pure oral arsenic trioxide that was invented and patented in Hong Kong. With memories of the Fowler’s solution, an oral As_2_O_3_ formulation or the “modern” liquor arsenicalis was revived in 1998 in Hong Kong. In Hong Kong, China, an oral preparation of As_2_O_3_ (oral-As_2_O_3_) was developed, and was shown to be efficacious for APL in first relapse (R1), inducing second complete remission (CR2) in more than 90% of patients (5). Further, in an effort to prevent relapses, oral-As_2_O_3_ was used during induction and maintenance of CR1 (6, 7). This strategy resulted in favorable overall-survival (OS) and leukemia-free-survival (LFS). This 1mg/ml oral-As_2_O_3_ solution has a bioavailability comparable with that of i.v. As_2_O_3_ (8). Oral arsenic trioxide (Arsenol ^®^) from Hong Kong was also the first oral preparation of pure arsenic trioxide produced under the Good Manufacturing Practice (GMP) standards. On the other hand, Realgar-Indigo naturalis formula (RIF) was developed in the 1980s entirely based on traditional Chinese Medicine (TCM) concepts and comprises realgar, *Indigo naturalis*, *Salvia miltiiorrhiza* and *Radix pseudostellariae* (9). Tetraarsenic tetrasulphide (As_4_S_4_), indirubin and tanshinone IIA are the active ingredients of RIF with *in-vitro* synergism (9). In this Research Topic, Zhu and Lou et al. described the all oral, chemotherapy-free model in the frontline management of APL highlighting the applications of RIF and summarized the clinical data and excellent outcome of patients treated with RIF-based induction. Zhu also highlighted a major obstacle to cure in APL, that is, early deaths. In addition, multi-centre clinical trials in patients treated with ATRA, arsenic trioxide and anthracyclines reported a relatively low incidence of early deaths of 3-10% (1, 2). Xu and Huang discuss the evolution of various therapeutic approaches from hospital-based induction and consolidation to home-based oral As_2_O_3_-based therapy especially during consolidation and maintenance. Other oral formulations of As_2_O_3_ are also being investigated with the hope of eventually developing an all oral, effective regimen in standard-risk APL (10).

With regard to the therapeutic application of intravenous arsenic trioxide, Russell and Dillon summarize the United Kingdom’s NCRI AML17 experience using the attenuated arsenic trioxide regimen in newly diagnosed APL. The treatment of “high-risk” APL remains a topic of contention as As_2_O_3_ is not yet approved for this indication. An important exploratory study by the MD Anderson Cancer Center using ATRA-As_2_O_3_ with or without gemtuzumab ozogamacin (GO) suggested that an essentially chemotherapy-free regimen might be feasible for the upfront treatment of APL (11). The NCRI AML17 trial built on these findings to investigate the de-intensification of treatment by randomizing patients irrespective of their risk status between As_2_O_3_-ATRA and the ATRA-idarubicin (AIDA) regimen. The AML17 trial included a total of 57 high-risk patients and their overall survival at 4 years was not significantly different from standard risk patients, being 95% in standard-risk compared with 87% in high-risk patients. Of the 28 high-risk patients in the As_2_O_3_ arm of AML17 who received the planned induction of ATRA, As_2_O_3_ and GO, the 4-year survival was 89% (12).

Last, but not least, Sanz et al. reappraised the role of hematopoietic stem cell transplantation (HSCT) in the current era of arsenic trioxide. As_2_O_3_-based regimens are currently regarded as the first option for relapsed APL. The selection of the most appropriate post-remission treatment option for patients in second CR (CR2), including the use of HSCT, depends on several variables, such as pre-transplant molecular status, duration of first remission, age, and donor availability. Despite modest evidence from retrospective studies, autologous HSCT has been the preferred option for consolidation in patients achieving molecularly negative CR2. The suitability of HSCT as compared with other non-HSCT alternatives has recently engendered much interest, thereby necessitating prospective controlled studies to define the role of HSCT in APL.

With the above backdrop, this special Research Topic provides an overview on the biology of APL and highlights perspectives on the past, present, and future treatment approaches in APL. This topic plays an important role in addressing the development of As_2_O_3_ –based therapy in improving the outcome of APL, from once the most fatal to currently the most curable leukemia.

## Author Contributions

HG wrote and approved the manuscript. All authors contributed to the article and approved the submitted version.

## Conflict of Interest

The University of Hong Kong currently holds two United States (US) patents (7,521,071 B2 and 8,906,422 B2), one Japanese patent (4786341) and one European patent (EP 1562616 B1) for the use of oral arsenic trioxide in the treatment of leukemias and lymphomas. HG is employed by the University of Hong Kong.

The remaining authors declare that the research was conducted in the absence of any commercial or financial relationships that could be construed as a potential conflict of interest.

## Publisher’s Note

All claims expressed in this article are solely those of the authors and do not necessarily represent those of their affiliated organizations, or those of the publisher, the editors and the reviewers. Any product that may be evaluated in this article, or claim that may be made by its manufacturer, is not guaranteed or endorsed by the publisher.
